# Effects of Previous Kasai Surgery on Gut Microbiota and Bile Acid in Biliary Atresia With End-Stage Liver Disease

**DOI:** 10.3389/fmed.2021.704328

**Published:** 2021-09-27

**Authors:** Wei Song, Li-Ying Sun, Zhi-Jun Zhu

**Affiliations:** ^1^Liver Transplantation Center, National Clinical Research Center for Digestive Diseases, Beijing Friendship Hospital, Capital Medical University, Beijing, China; ^2^Clinical Center for Pediatric Liver Transplantation, Capital Medical University, Beijing, China; ^3^Department of Intensive Care Unit, Beijing Friendship Hospital, Capital Medical University, Beijing, China

**Keywords:** biliary atresia, Kasai surgery, bile acids, gut microbiota (GM), metagenomic sequencing

## Abstract

**Background and Aims:** Biliary atresia (BA) is the most common cholestatic liver disease in neonates. Although the Kasai procedure can improve temporary biliary drainage in some cases, complications and liver fibrosis still develop. Liver transplantation is the ultimate treatment. The current study aimed to investigate the effect of previous Kasai surgery on gut microbiota and bile acid in BA with end-stage liver disease.

**Methods:** Patients with BA with end-stage liver disease were divided into two groups according to whether they had previously undergone Kasai surgery (non-Kasai: *n* = 8, post-Kasai: *n* = 8). Metagenomic sequencing and ultraperformance liquid chromatography/tandem mass spectrometry were performed to identify the gut microbiota and bile acid.

**Results:** Previous Kasai surgery had some effects on gut microbiota and bile acid in BA with end-stage liver disease. In the gut microbiome, the differential species were mainly distributed at the species level. *Veillonella atypica* had a significant increase in the non-Kasai group (*P* < 0.05). *Bacteroides* spp., *Prevotella* spp., *Barnesiella* spp., *Parabacteroides* spp., *Heliobacterium* spp., *Erysipelatoclostridium* spp. and *Diaporthe* spp. were increased in the post-Kasai group (*P* < 0.05). Concerning functional profiles, methionine biosynthesis was enriched in the non-Kasai group, while pyridoxal biosynthesis and riboflavin biosynthesis were enriched in the post-Kasai group (linear discriminant analysis > 2, *P* < 0.05). In stools, 17 bile acids were distinctly elevated in the post-Kasai group, such as cholic acid, chenodeoxycholic acid, β-muricholic acid and tauro α-muricholate (*P* < 0.05). Spearman correlation test showed that *V. atypica* had an enormously positive correlation with liver enzymes. *Faecalibacterium prausnitzii* and *Escherichia coli* were associated with derivatives of the alternative pathway of bile acid metabolism.

**Conclusion:** Previous Kasai surgery can improve the gut microbiota and bile acid in patients with BA with end-stage liver disease. This improvement contributes to maintaining the intestinal barrier.

## Introduction

Biliary atresia (BA) is the most common cause of cholestatic liver disease in children. It is characterized by intrahepatic and extrahepatic bile duct occlusion and bile drainage obstruction (1, 2). It is fatal if left untreated, with a reported survival rate of <10% at 3 years of age (3). Kasai portoenterostomy (KPE) is considered the primary treatment of BA, but its outcome is still unsatisfactory (4). Although this approach can improve the short-term work, most patients develop fibrosis and progress to end-stage liver disease (5). Liver transplantation is still the only available salvage treatment. It is indicated when: (1) the Kasai procedure fails; (2) patients develop progressive deterioration of liver function despite an initially successful Kasai operation; or (3) end-stage liver disease develops in children who have not undergone Kasai surgery (6).

BA is a rare disease with an unclear etiology. There is strong evidence that viruses and toxins contribute to BA. Cytomegalovirus, human papillomavirus, human herpesvirus 6, Epstein–Barr virus, reovirus, and rotavirus have been detected directly in injured liver and biliary remnants, or indirectly by the presence of serological markers of infection in patients with BA (7). Viruses trigger an inflammatory response that injures the duct epithelium and produces rapidly progressive cholangiopathy. As for disease progression, Isaacs-Ten et al. have demonstrated that exposure to bacterial endotoxin sensitizes liver cells to bile-acid-induced cell death in cholestatic liver disease (8). When the intestinal barrier is damaged, translocated bacteria and microbial toxins can enter the portal circulation and access the liver (9). Therefore, there is a special relationship between the gut microbiota and liver injury.

The human gut microbiota composition can be altered by multiple factors such as diet, age, antibiotics, and various diseases. Among these, bile acids appear to be significant regulators. Bile acids interact with gut microbiota through the gut–liver axis (10). Bile acids can affect the composition of gut microbes by controlling the pH of the gut environment, inhibiting the growth of pathogens, and maintaining the balance of gut microbes (11). Decreased bile acids in the gut contribute to the overgrowth of potential pathogens, many of which produce lipopolysaccharide (LPS). Conversely, increased bile acids favor growth of Gram-positive Firmicutes (12).

The gut microbiota participates in and influences the enterohepatic circulation of bile acids. The conversion of primary to secondary bile acids depends on the bile salt hydrolase on the surface of certain bacteria in the gut (13–15). Previous research has reported that bacterial dysbiosis is linked to low bile acid levels entering the intestine in cirrhosis (16). There is still some unknown crosstalk between specific microbiota and bile acid metabolism that needs to be explored.

In the current study, we aimed to investigate the effect of previous Kasai surgery on the gut microbiome and bile acids in patients with BA with end-stage liver disease, then explore their relationship and its impact on health. High-throughput techniques, such as 16S rRNA genes and metagenomic sequencing, compared to traditional culture-dependent methods, have significantly improved the ability to rapidly determine the composition of the gut microbiome and its functions (17). The present study combined 16S rRNA genes with subsequently metagenomic sequencing to make the results more reliable.

## Materials and Methods

### Study Design and Sample Collection

We recruited patients with BA listed for liver transplantation at Beijing Friendship Hospital, Capital Medical University, between September 2017 and December 2018. The diagnosis in these patients was previously confirmed by laparotomy or operative cholangiography. Enrolled patients had to meet the following criteria: (1) age <3 years; (2) diagnosed with type III BA; (3) no antibiotics or probiotics within 1 month; (4) no digestive diseases such as diarrhea or constipation; and (5) similar dietary habits. Differential diagnoses were excluded, including bile duct dysplasia, progressive familial intrahepatic cholestasis, citrin deficiency disease, tyrosinemia type 1, and α-1 antitrypsin deficiency. The patients were divided into two groups based on whether they had previously undergone Kasai surgery. Each group consisted of eight patients (the non-Kasai and post-Kasai groups). The detailed demographic data and clinical indicators are shown in Table 1.

**Table 1:** Clinical characteristics of the patients.

	**Non-Kasai (*n* = 8)**	**Post-Kasai (*n* = 8)**	***P*-value**
Age (months), median (range)	7.5 (4.8–9.8)	10.95 (5.5–21.0)	0.0463
Sex			NS
Female, n (%)	5 (62.5%)	5 (62.5%)	
Male, n (%)	3 (37.5%)	3 (37.5%)	
BMI, kg/m^2^, median (range)	17.75 (11.06–19.33)	16.06 (12.26–21.46)	NS
Hepatic function, median (range)	
ALT, U/L	172.5 (119.00–439.00)	74.5 (21.00–177.00)	0.0379
AST, U/L	353.7 (221.40–724.30)	120.1 (45.40–202.40)	0.0002
ALP, U/L	748 (372.00–1409.00)	516.5 (296.00–809.00)	NS
GGT, U/L	357.5 (26.00–980.00)	180.5 (50.00–327.00)	NS
TBA, μmol/L	184.9 (71.10–355.1)	139.9 (9.02–659.14)	NS
TBIL, μmol/L	513.8 (260.76–898.13)	77.6 (57.00–356.00)	0.0499
NH3, μmol/L	103.13 (47.00–154.00)	70.88 (48.00–257.00)	NS
Vit D, ng/ml, median (range)	5.92 (3.00–15.33)	6.48 (4.23–14.76)	NS
PELD, median (range)	29 (21.00–35.00)	13.5 (1.00–39.00)	NS

*ALT, alanine aminotransferase; AST, aspartate aminotransferase; ALP, alkaline phosphatase; GGT, γ-glutamyltransferase; TBA, total bile acids; TBIL, total bilirubin; NH3, ammonia; Vit D, Vitamin D; PELD, pediatric end-stage liver disease*.

### Sample Collection and DNA Extraction

All samples were stored at −80°C within 4 h of collection. Bacterial DNA was extracted using the QIAamp Fast DNA Stool Mini Kit (51604; Qiagen, Hilden, Germany). Ten micrograms of stool sample were weighed in a centrifuge tube, approximately 25 mg of precooled submerged beads were added, and 200 μl acetonitrile/methanol (v/v = 8:2) solvent containing 10 μl internal standard for homogeneous mixing was added and centrifuged at 13,500 rpm and 4°C for 20 min to remove proteins. After centrifugation, 10 μl supernatant was obtained, diluted with 90 μl 1:1 acetonitrile/methanol (v/v = 80/20) and ultrapure water mixed solvent, shaken and centrifuged for analysis. The injection volume was 5 μL. The DNA concentration was measured with a NanoDrop (Thermo Scientific, MA, USA) and Qubit®2.0 (Invitrogen, Carlsbad, CA, USA), and the molecular size was estimated by agarose gel electrophoresis.

### Library Construction and Metagenomic Sequencing

Following the Illumina TruSeq DNA Sample Prep v2 Guide (San Diego, CA, USA), we constructed the DNA paired-end libraries with an insert size of 500 bp for the 40 stool samples (16 from pre-LT, 14 from post-LT, and 10 from controls). The quality of all libraries was evaluated using an Agilent 2100 bioAnalyzer (Agilent Technologies, Wokingham, UK) and an Agilent 2100 DNA 1000 kit. All samples were subjected to 150-bp paired-end sequencing on an Hiseq X-ten platform (Illumina). Illumina raw reads were screened according to the following criteria: (1) adaptor contamination reads were removed; (2) reads containing more than three ambiguous N bases were removed; (3) reads containing low quality (Q <20) bases were trimmed; and (4) reads containing <60% of high-quality bases (Phred score ≥20) were deleted.

Clean reads were subjected to bacterial genomes from the National Center for Biotechnology Information GenBank with SOAPaligner (version 2.21), and reads mapped to the host genome were abandoned.

For species classification, the NCBI database (http://www.ncbi.nlm.nih.gov) was used to align the clean reads with known bacteria, fungi, viruses, and archaea by SOAPaligner 2.21. As to the functional profiles, the non-redundant genes were annotated against the Kyoto Encyclopedia of Genes and Genomes (KEGG) database using BLAST (v. 2.2.28+). When the assembled protein sequence was similar (score ≥60 and E <1 × 10^−5^) to a protein sequence in the database, the assembled protein was considered to play the same role as the database protein. The relative abundance of all orthologous genes was accumulated to generate the close lot of each KEGG ortholog. The results of metagenomic sequencing and assembly data in each sample are listed in Supplementary Table 1.

### Quantitative Determination of Stool Bile Acid Spectrum

#### Reagents and Instruments

Bile acid standards (Steraloids, USA), six stable isotopes labeled standards (C/D/NIsotopes, Canada/Steraloids), ammonium acetate (analytical grade, Sigma–Aldrich, St Louis, MO, USA), methanol (Optima LC-MS), acetonitrile (Optima LC-MS), isopropanol (Optima LC-MS), glacial acetic acid and formic acid (Optima LC-MS) were all purchased from ThermoFisher Scientific (Fairlawn, NJ, USA). The following equipment was used: ACQUITYUPLC-XevoTQ-S liquid-mass spectrometer (WatersCorp., Milford, MA, USA); Mill-Q ultrapure water system (Millipore, Billerica, MA, USA); homogenizer (BB24; NextAdvance, Averill Park, NY, USA); microcentrifuge (Microfuge20R; Beckman Coulter, Indianapolis, IN, USA); and lyophilizer (Labconco, Kansas City, MO, USA).

#### Experimental Method

Seventy-three bile acid standards were used, and six representative isotope bile acids were used as internal standards for calibration. Standards and isotope markers were accurately weighed and prepared with methanol to a concentration of 5.0 mM. We mixed the standards in serum matrix without bile acids and set seven concentrations of 2000, 1000, 400, 100, 25, 10 and 5 nM. We weighed 10 mg stool sample in a centrifuge tube, added ~25 mg of precooled submerged beads, and 200 μl acetonitrile/methanol (v/v = 8:2) solvent containing 10 μl internal standard for homogeneous mixing, centrifuged at 13,500 rpm and 4°C for 20 min to remove protein. After centrifugation, 10μl supernatant was diluted with 90 μl 1:1 acetonitrile/methanol (80/20) and ultrapure water mixed solvent, shaken and centrifuged before injection analysis. The injection volume was 5μl. Ultra-high performance liquid chromatography-tandem mass spectrometry (UPLC-MS/MS) system (ACQUITYUPLC-XevoTQ-S; Waters) was used for quantification of metabolites (18).

### Statistical Analysis

The non-parametric Wilcoxon test (Wilcox. test in R) was performed to analyze the statistical significance of the different taxonomic levels between the different cohorts. Differences were considered significant at *P* < 0.05 or false discovery rate (FDR) <0.1. Linear discriminant analysis (LDA) effect size (LEfSe) analysis was used to identify the taxa most likely to explain differences between the post-Kasai and non-Kasai groups. The LDA score cut-off of 2.0 indicated a significant difference. Orthogonal partial least squares discriminate analysis (OPLS-DA) was used for statistical analysis to determine stool bile acid changes between the two groups. All the metabolite variables were scaled to pareto scaling prior to conducting the OPLS-DA. The model validity was evaluated from model parameters R2 and Q2, which provided information for the interpretability and predictability, respectively, of the model and avoided the risk of overfitting. Variable importance in the projection (VIP) was calculated in the OPLS-DA model. The VIP score cut-off of 1.0 indicated a significant difference. The Spearman correlation test was conducted to investigate the relationship between the clinical parameters, bile acid, and microbial composition. A heat map was drawn using the R software corrplot package/gplots package to illustrate the results.

## Results

### Differential Intestinal Microbiota Between Post-Kasai and Non-Kasai Groups

**Figure 1:**
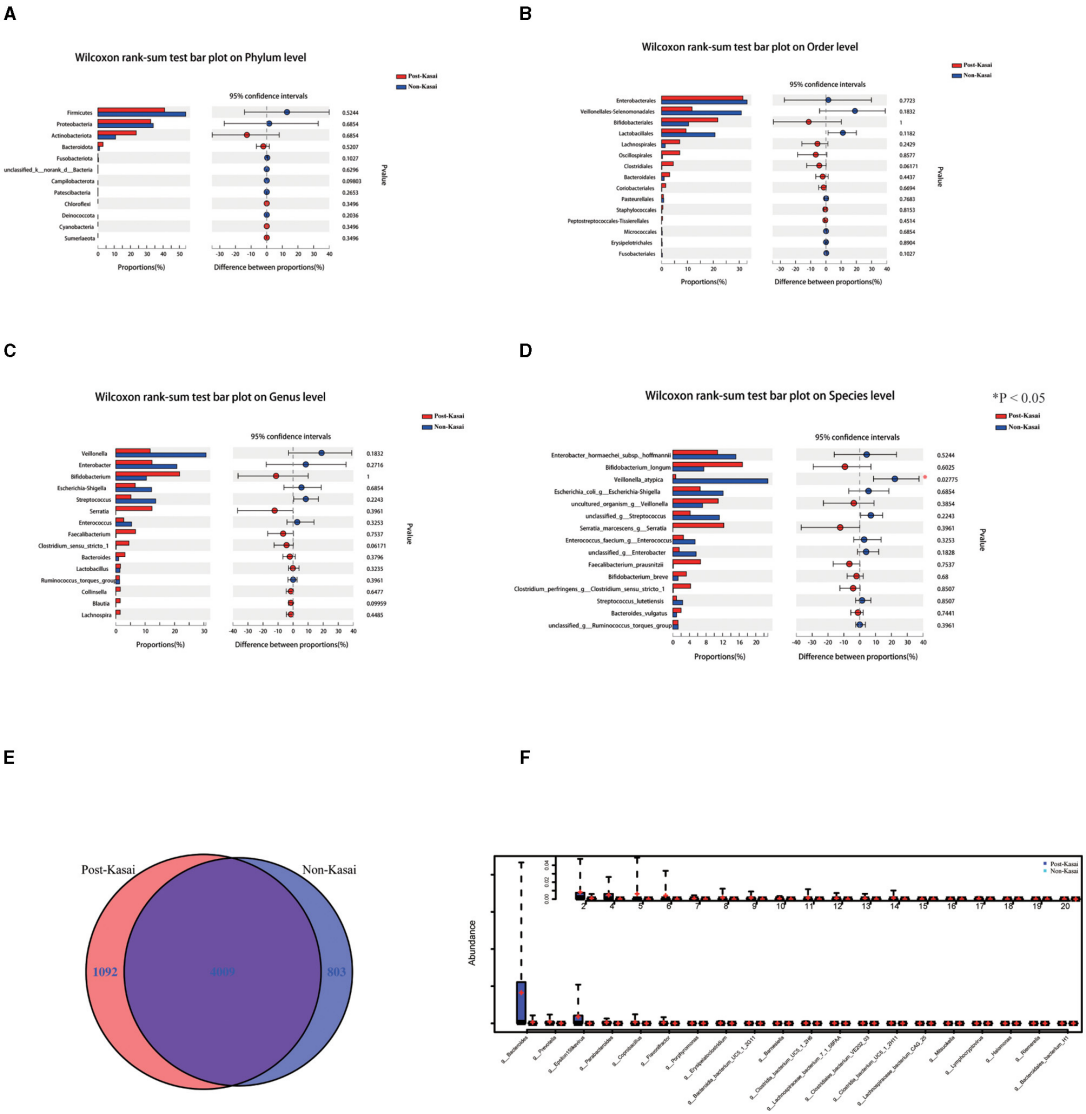
**(A–D)** Differential gut microbiota between the post-Kasai and non-Kasai groups at the phylum, order, genus and species levels (*P < 0.05, 16S rRNA genes). **(E)** Venn diagram of the post-Kasai and non-Kasai groups at the species level. **(F)** Differential gut microbiota between the post-Kasai and non-Kasai groups performed by metagenomic sequencing.

16S rRNA gene sequencing was performed to determine the alterations in the gut microbiota between the two groups. It showed no significant difference at the phylum, order or genus level (Figures 1A–C). *Shigella, Streptococcus* and *Enterococcus* abundances were higher in the non-Kasai group although they did not reach statistical significance (*P* > 0.05, Supplementary Table 2). However, *Veillonella atypica* had a noticeable increase in the non-Kasai group at the species level (Figure 1D, *P* < 0.05) (Supplementary Table 3).

**Figure 2:**
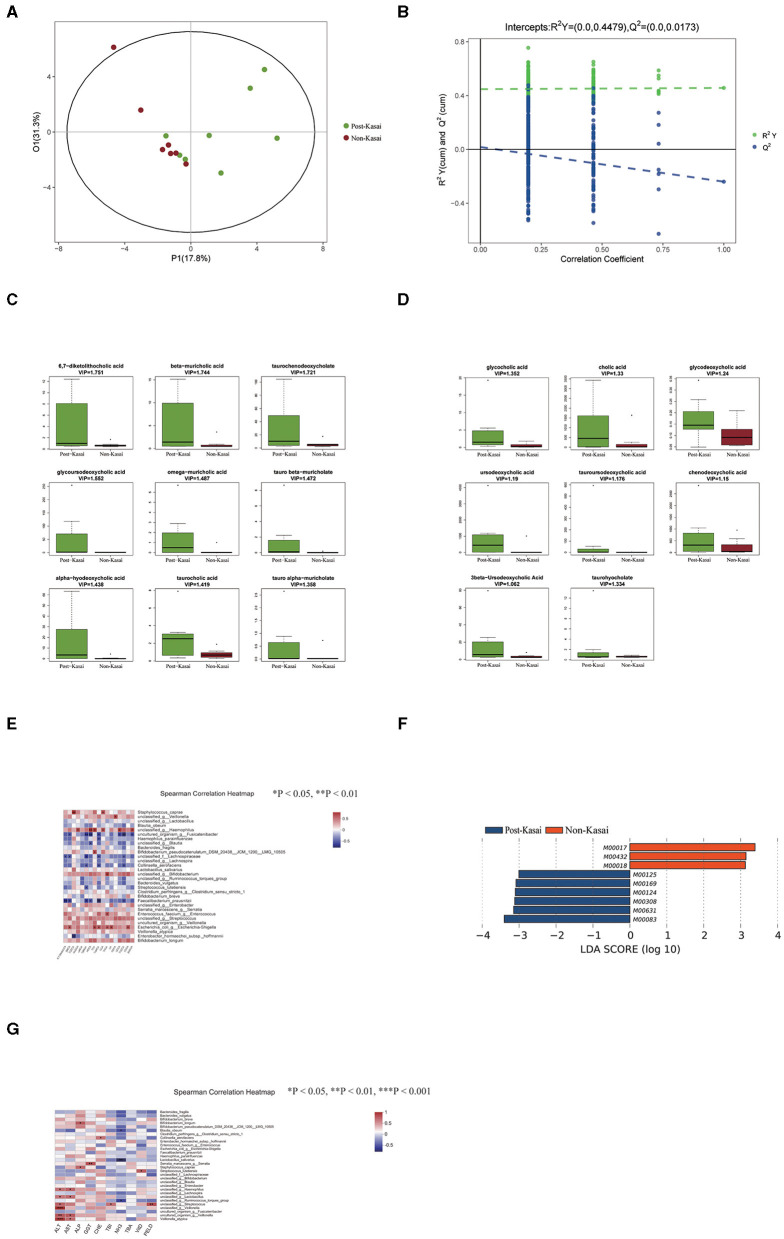
**(A,B)** Significant differences in stool bile acid composition between the post-Kasai and non-Kasai groups identified by OPLS-DA (left: OPLS-DA map; right: model verification map OPLS-DA). Model verification map of OPLS-DA: the x axis represents the replacement retention of the replacement test; the y axis represents the R2 (green dot) and Q2 (blue dot) replacement test values. The two dashes represent the regression lines of R2 and Q2, respectively. **(C,D)** Differential bile acids between the two groups. The x axis represents the different groups, and the y axis expression of screened differential bile acids. Different colors represent different groups, and the boxplot shows five statistical values (minimum, first quartile, median, third quartile, and maximum, namely five lines from bottom to top). **(E)** Spearman correlations between gut species and bile acids. The x axis represents the differential bile acids, and the y axis the species (*P < 0.05, **P < 0.01, ***P < 0.001). Blue denotes a negative correlation and red a positive correlation. **(F)** Differential functional profiles between the two groups. **(G)** Spearman correlations between gut species and clinical indicators (*P < 0.05, **P < 0.01, ***P < 0.001). The x axis represents the environmental factors, and the y axis the species. Blue denotes a negative correlation and red a positive correlation.

Metagenomic sequencing was used further to identify the differential species between the two groups. There were 803 and 1,092 species enriched in the non-Kasai and post-Kasai groups, respectively (Figure 1E). We concluded that Kasai surgery increased the diversity of species in BA. *Bacteroides, Prevotella, Barnesiella, Parabacteroides, Heliobacterium, Erysipelatoclostridium* and *Diaporthe* were increased in the post-Kasai group (Figure 1F, Supplementary Table 4). Spearman correlation test showed that the abundance of *Veillonella* spp. (e.g., *V. atypica*) was strongly positively correlated with liver enzyme alanine aminotransferase (ALT) and aspartate aminotransferase (AST), but had no significant correlation with total bile acid (Figure 2G, Supplementary Table 5). Therefore, we speculated that *V. atypica* contributed to the liver injury in BA.

### Alteration of Bile Acids Between the Post-Kasai and Non-Kasai Groups

A total of 46 fecal bile acids were detected, and OPLS-DA was used to screen for differential metabolites between the two groups (Figures 2A,B, permutation test: R^2^Y = 0.0–0.4479, Q^2^ = 0.0–0.0173). Seventeen bile acid levels were significantly elevated in the post-Kasai group (Figures 2C,D, VIP > 1) (Supplementary Table 6).

In the increased bile acid, β-muricholic acid (βMCA), ω-muricholic acid (ωMCA), tauro α-muricholate (TαMCA), tauro β-muricholate (TβMCA), taurohyocholate (THCA), and α-hyodeoxycholic acid (HDCA) belonged to the products of the alternative pathway, and the remaining bile acids were the products of the classical pathway. Spearman correlation test was subsequently conducted to investigate the relationship between the differential bile acids and species (Figure 2E, Supplementary Table 7). The level of βMCA, TαMCA, TβMCA and HDCA was strongly negatively correlated with the abundance of *Faecalibacterium prausnitzii* but positively correlated with *Escherichia coli*. Importantly, these bile acids were all derived from the alternative pathway. Previous research has shown that the classical pathway of bile acid metabolism is impaired, while the alternative pathway is preserved in infantile cholestasis (19). We inferred that the altered abundance of *F. prausnitzii* and *E. coli* contributed to the changed bile acid metabolism in BA.

### Differential Functional Profiles Between the Post-Kasai and Non-Kasai Groups

We annotated the catalogs using the KEGG database to investigate the gut microbiome's functional profiles (http://www.genome.jp/kegg/). There were nine differential functional modules between the post-Kasai and non-Kasai groups (Figure 2F). Six were enriched in the post-Kasai group and the remaining three were in the non-Kasai group (Supplementary Table 8, *P* < 0.05). Methionine biosynthesis (M00017) was enriched in the non-Kasai group, while pyridoxal biosynthesis and riboflavin biosynthesis (M00124 and M00125) were enriched the post-Kasai group.

## Discussion

As the largest immune organ in the human body, intestinal microbes play an essential role in multiple physiological functions and are called the second human genome (20). Mounting evidence supports an association between liver disease and changes in the microbiome composition (16, 21–25). However, the role of previous Kasai surgery in the gut microbiota and bile acids in patients with BA with the end-stage liver disease remains largely undefined. An in-depth understanding of the gut microbiota is essential for exploring the relationship between the microbiome and disease pathogenesis. We, therefore, used 16S rRNA and metagenomic sequencing to analyze and identify fecal microorganisms.

Compared to 16S rRNA sequencing, metagenomic sequencing identified more differential species between the non-Kasai and post-Kasai groups. Overall, the number of species was greater in the post-Kasai group, attributed to bile acid drainage. Differences in microbiota composition are mainly at the species level, and in particular, *V. atypica* showed a significant decrease in the post-Kasai group. *Veillonella* is reported to be associated with autoimmune liver disease (22). Correlation analysis showed that *V. atypica* was positively correlated with liver enzymes. The above data suggested that the abundance of *V. atypica* contributed to liver injury in BA. Besides, *Bacteroides, Prevotella, Clostridium* spp., *Barnesiella, Parabacteroides, Heliobacterium, Erysipelatoclostridium* and *Diaporthe* were also enriched in the post-Kasai group. *Clostridium* spp., *Barnesiella* and *Prevotellaceae* contain genes that produce the short-chain fatty acids, propionate and butyrate, which play important roles in maintaining the intestinal barrier and anti-inflammation (26). *Bacteroides* spp. have been associated with health (9). Consistent with these results, methionine biosynthesis was decreased in the post-Kasai group. Previous research has demonstrated that dietary methionine restriction improves the gut microbiota and reduces intestinal permeability and inflammation (27). We concluded that the gut microbiota, intestinal permeability, and inflammation were improved in the post-Kasai group.

Bile acids are synthesized in the liver by multistep reactions catalyzed via two distinct routes, the classical and alternative pathways (28). The classical pathway is initiated by the rate-limiting enzyme cholesterol 7α-hydroxylase (CYP7A1) and results in the formation of the primary BAs, CA and CDCA. The alternative pathway is initiated with the oxidation of the cholesterol side-chain by the mitochondrial cytochrome p450 sterol 27-hydroxylase (CYP27A1) followed by 25-hydroxycholesterol 7-alpha-hydroxylase (CYP7B1) (29). HCA, αMCA, βMCA, and their conjugated bile acids are the products of this pathway. The classical pathway accounts for about 75% of bile acid production. The gut microbiome harbors hundreds of pathways, many of which modulate host biology. In the intestine, bile acids are subject to extensive metabolism by gut microbes, namely deconjugation of glycine or taurine and biotransformation of the unconjugated primary bile acids to secondary bile acids (30). Deoxycholic acid, lithocholic acid (LCA) and its derivatives are major components of the recirculating bile acid pool (31). Consistently, 6,7-diketolithocholic acid (6,7-DiketoLCA), one derivative of LCA, was increased in the post-Kasai group. Previous research has demonstrated that disorder of bile acid metabolism is related to inflammatory bowel disease (32). We observed that the abundance of *F. prausnitzii* and *E. coli* was related to the alternative pathway of bile acid metabolism.

As for functional profiles, it was observed that the pathway of pyridoxal and riboflavin biosynthesis was higher in the post-Kasai group. Pyridoxal is one of the pyridine derivatives from vitamin B6. Vitamin B6 deficiency affects cell-mediated immunity in both animal and human studies (33). Riboflavin (vitamin B2) is unique among water-soluble vitamins. There are reports of various congenital malformations associated with riboflavin deficiency in rats and mice. Besides, riboflavin synthesized by bacterial metabolism in the colon might be a more critical source (34). Based on functional results, it appeared that the post-Kasai group was healthier although it still needs verification by microbial metabolomics.

This study had some limitations. (1) The number of patients was small, and a greater number of patients should be enrolled. We will expand the sample size in future research to verify these results. (2) Alterations in the functional microbiome profiles and the correlations between the gut microbiome, bile acid, and clinical indicators need to be verified. Microbial metabolomics will be performed to understand their functions and correlations deeply.

In conclusion, 16S rRNA and metagenomic sequencing revealed that previous Kasai surgery can improve the gut microbiota composition in patients with BA with end-stage liver disease. *V. atypica* was decreased while *Bacteroides, Prevotella, Barnesiella, Parabacteroides, Heliobacterium, Erysipelatoclostridium* and *Diaporthe* were increased in the post-Kasai group. *V. atypica* might contribute to liver injury in BA. UPLC-MS/MS was performed to detect characteristic changes in stool bile acids. We conclude that the abundance of *F. prausnitzii* and *E. coli* is related to the alternative pathway of bile acid metabolism.

## Data Availability Statement

The datasets presented in this study can be found in online repositories. The names of the repository/repositories and accession number(s) can be found below: NCBI SRA (https://www.ncbi.nlm.nih.gov/bioproject PRJNA730640), it can be accessed with the BioProject identifier PRJNA730640.

## Ethics Statement

The studies involving human participants were reviewed and approved by Beijing Friendship Hospital, Capital Medical University (Approval ID: 2019-P2–131-02). Written informed consent to participate in this study was provided by the participants' legal guardian/next of kin.

## Author Contributions

WS: study design, data collection, analysis and interpretation of the data, and writing of the report. L-YS and Z-JZ: study design, study supervision, and critical revision of the manuscript for important intellectual content. All authors have read and approved the final manuscript to be submitted.

## Funding

This study was supported by the National Natural Science Foundation of China (Grant No. 81570586).

## Conflict of Interest

The authors declare that the research was conducted in the absence of any commercial or financial relationships that could be construed as a potential conflict of interest.

## Publisher's Note

All claims expressed in this article are solely those of the authors and do not necessarily represent those of their affiliated organizations, or those of the publisher, the editors and the reviewers. Any product that may be evaluated in this article, or claim that may be made by its manufacturer, is not guaranteed or endorsed by the publisher.

## Abbreviations

BA: biliary atresia
ALT: alanine aminotransferase
AST: aspartate aminotransferase
ALP: alkaline phosphatase
GGT: γ-glutamyltransferase
TBA: total bile acids
TBIL: total bilirubin
PELD: pediatric end-stage liver disease
KEGG: Kyoto encyclopedia of genes and genomes
LEfSe: LDA effect size
LDA: linear discriminant analysis
βMCA: β-muricholic acid
ωMCA: ω-muricholic acid
TαMCA: Tauro α-muricholate
TβMCA: Tauro β-muricholate
THCA: taurohyocholate
HDCA: alpha-hyodeoxycholic acid
CDCA: chenodeoxycholic acid
CA: cholic acid
GCA: glycocholic acid
TCDCA: taurochenodeoxycholic acid
GUDCA: glycoursodeoxycholic acid
TCA: taurocholic acid
UDCA: ursodeoxycholic acid
GDCA: glycodeoxycholic acid
TUDCA: tauroursodeoxycholic acid
6, 7-DiketoLCA, 6: 7-diketolithocholic acid
βUDCA: 3β-Ursodeoxycholic Acid
OPLS-DA: orthogonal partial least squares discrimination analysis
VIP: variable importance in the projection.

## References

[1] Lakshminarayanan B Davenport M. Biliary atresia: a comprehensive review. J Autoimmun. (2016) 73:1–9. 10.1016/j.jaut.2016.06.005 27346637

[2] Vij M Rela M. Biliary atresia: pathology, etiology and pathogenesis. Future Sci OA. (2020) 6:FSO466. 10.2144/fsoa-2019-0153 32518681 PMC7273417

[3] Siddiqui AI Ahmad T. Biliary Atresia. Treasure Island, FL, StatPearls. (2020). 30725947

[4] Chung PHY Zheng S Tam PKH. Biliary atresia: east versus west. Semin Pediatr Surg. (2020) 29:150950. 10.1016/j.sempedsurg.2020.150950 32861448

[5] Sundaram SS Mack CL Feldman AG Sokol RJ. Biliary atresia: Indications and timing of liver transplantation and optimization of pretransplant care. Liver Transpl. (2017) 23:96–109. 10.1002/lt.24640 27650268 PMC5177506

[6] Wang SH Chen CL Concejero A Wang CC Lin CC Liu YW. Living donor liver transplantation for biliary atresia. Chang Gung Med J. (2007) 30:103–8. 10.1016/j.transproceed.2010.11.002 17595997

[7] Asai A Miethke A Bezerra JA. Pathogenesis of biliary atresia: defining biology to understand clinical phenotypes. Nat Rev Gastroenterol Hepatol. (2015) 12:342–52. 10.1038/nrgastro.2015.74 26008129 PMC4877133

[8] Isaacs-Ten A Echeandia M Moreno-Gonzalez M Brion A Goldson A Philo M. Intestinal microbiome-macrophage crosstalk contributes to cholestatic liver disease by promoting intestinal permeability in mice. Hepatology. (2020) 72:2090–108. 10.1002/hep.31228 32168395 PMC7839474

[9] Chopyk DM Grakoui A. Contribution of the intestinal microbiome and gut barrier to hepatic disorders. Gastroenterology. (2020) 159:849–63. 10.1053/j.gastro.2020.04.077 32569766 PMC7502510

[10] Tripathi A Debelius J Brenner DA Karin M Loomba R Schnabl B. The gut-liver axis and the intersection with the microbiome. Nat Rev Gastroenterol Hepatol. (2018) 15:397–411. 10.1038/s41575-018-0011-z 29748586 PMC6319369

[11] Long SL Gahan CGM Joyce SA. Interactions between gut bacteria and bile in health and disease. Mol Aspects Med. (2017) 56:54–65. 10.1016/j.mam.2017.06.002 28602676

[12] Ridlon JM Kang DJ Hylemon PB Bajaj JS. Bile acids and the gut microbiome. Curr Opin Gastroenterol. (2014) 30:332–8. 10.1097/MOG.0000000000000057 24625896 PMC4215539

[13] Yang X Lu D Zhuo J Lin Z Yang M Xu X. The gut-liver axis in immune remodeling: new insight into liver diseases. Int J Biol Sci. (2020) 16:2357–66. 10.7150/ijbs.46405 32760203 PMC7378637

[14] Woodhouse C Singanayagam A Patel VC. Modulating the gut-liver axis and the pivotal role of the faecal microbiome in cirrhosis. Clin Med. (2020) 20:493–500. 10.7861/clinmed.2020-0676 32934044 PMC7539737

[15] Wang F Cui Q Zeng Y Chen P. [Gut microbiota-an important contributor to liver diseases]. Nan Fang Yi Ke Da Xue Xue Bao. (2020) 40:595–600. 10.12122/j.issn.1673-4254.2020.04.23 32895142 PMC7225115

[16] Qin N Yang F Li A Prifti E Chen Y Shao L. Alterations of the human gut microbiome in liver cirrhosis. Nature. (2014) 513:59–64. 10.1038/nature13568 25079328

[17] Human Microbiome Project C. A framework for human microbiome research. Nature. (2012) 486:215–21. 10.1038/nature11209 22699610 PMC3377744

[18] Xie G Zhong W Li H Li Q Qiu Y Zheng X. Alteration of bile acid metabolism in the rat induced by chronic ethanol consumption. FASEB J. (2013) 27:3583–93. 10.1096/fj.13-231860 23709616 PMC3752538

[19] Wang Y Gao X Zhang X Xiao Y Huang J Yu D. Gut Microbiota dysbiosis is associated with altered bile acid metabolism in infantile cholestasis. mSystems. (2019) 4. 10.1128/mSystems.00463-19 31848302 PMC6918028

[20] Zhu B Wang X Li L. Human gut microbiome: the second genome of human body. Protein Cell. (2010) 1:718–25. 10.1007/s13238-010-0093-z 21203913 PMC4875195

[21] Abe K Fujita M Hayashi M Okai K Takahashi A Ohira H. Gut and oral microbiota in autoimmune liver disease. Fukushima J Med Sci. (2020) 65:71–5. 10.5387/fms.2019-21 31564673 PMC7012591

[22] Wei Y Li Y Yan L Sun C Miao Q Wang Q. Alterations of gut microbiome in autoimmune hepatitis. Gut. (2019) 69:569–77. 10.1136/gutjnl-2018-317836 31201284

[23] Tang R Wei Y Li Y Chen W Chen H Wang Q. Gut microbial profile is altered in primary biliary cholangitis and partially restored after UDCA therapy. Gut. (2018) 67:534–41. 10.1136/gutjnl-2016-313332 28213609

[24] Yu LX Schwabe RF. The gut microbiome and liver cancer: mechanisms and clinical translation. Nat Rev Gastroenterol Hepatol. (2017) 14:527–39. 10.1038/nrgastro.2017.72 28676707 PMC6467288

[25] Dubinkina VB Tyakht AV Odintsova VY Yarygin KS Kovarsky BA Pavlenko AV. Links of gut microbiota composition with alcohol dependence syndrome and alcoholic liver disease. Microbiome. (2017) 5:141. 10.1186/s40168-017-0359-2 29041989 PMC5645934

[26] Holota Y Dovbynchuk T Kaji I Vareniuk I Dzyubenko N Chervinska T. The long-term consequences of antibiotic therapy: Role of colonic short-chain fatty acids (SCFA) system and intestinal barrier integrity. PLoS ONE. (2019) 14:e0220642. 10.1371/journal.pone.0220642 31437166 PMC6705842

[27] Yang Y Zhang Y Xu Y Luo T Ge Y Jiang Y. Dietary methionine restriction improves the gut microbiota and reduces intestinal permeability and inflammation in high-fat-fed mice. Food Funct. (2019) 10:5952–68. 10.1039/C9FO00766K 31475718

[28] Jia W Wei M Rajani C Zheng X. Targeting the alternative bile acid synthetic pathway for metabolic diseases. Protein Cell. (2021) 12:411–25. 10.1007/s13238-020-00804-9 33252713 PMC8106556

[29] Rizzolo D Buckley K Kong B Zhan L Shen J Stofan M. Bile acid homeostasis in a cholesterol 7alpha-hydroxylase and sterol 27-hydroxylase double knockout mouse model. Hepatology. (2019) 70:389–402. 10.1002/hep.30612 30864232 PMC7893641

[30] Heinken A Ravcheev DA Baldini F Heirendt L Fleming RMT Thiele I. Systematic assessment of secondary bile acid metabolism in gut microbes reveals distinct metabolic capabilities in inflammatory bowel disease. Microbiome. (2019) 7:75. 10.1186/s40168-019-0689-3 31092280 PMC6521386

[31] Funabashi M Grove TL Wang M Varma Y McFadden ME Brown LC. A metabolic pathway for bile acid dehydroxylation by the gut microbiome. Nature. (2020) 582:566–70. 10.1038/s41586-020-2396-4 32555455 PMC7319900

[32] Duboc H Rajca S Rainteau D Benarous D Maubert MA Quervain E. Connecting dysbiosis, bile-acid dysmetabolism and gut inflammation in inflammatory bowel diseases. Gut. (2013) 62:531–9. 10.1136/gutjnl-2012-302578 22993202

[33] Ueland PM McCann A Midttun O Ulvik A. Inflammation, vitamin B6 and related pathways. Mol Aspects Med. (2017) 53:10–27. 10.1016/j.mam.2016.08.001 27593095

[34] Powers HJ. Riboflavin (vitamin B-2) and health. Am J Clin Nutr. (2003) 77:1352–60. 10.1093/ajcn/77.6.1352 12791609

